# Acute Infantile Bronchopneumonia

**Published:** 1902-09-27

**Authors:** 


### ACUTE INFANTILE BRONCHO-PNEUMONIA.

According to the standpoint from which we regard this condition, it may be considered as a disease in itself or as the cause of death in various other maladies. But in any case it is a disease which, from its frequency and fatality, must be of the greatest interest to all who are brought in contact with sick children. Dr. John Lindsay Steven [1] rightly protests against the various names by which this affection is commonly known, such as "acute lobular pneumonia," "acute broncho-pneumonia," or "acute capillary bronchitis," objecting more especially to the use of the term pneumonia, for, as he says, the disease is something quite different from pneumonia; and arguing from the fact that all the tissues of the lung are inflamed he suggests "acute infantile broncho-pneumonitis" as being a more appropriate designation. In regard to the question, Is this infantile broncho-pneumonitis one disease or many? Dr. Lindsay Steven shows that very different bacteria have been found in different cases, or groups of cases, and says that it is caused not by one (specific) but by many micro-organisms. Then as to the morbid anatomy, the most striking feature of the cases on which his remarks were founded was that the inflammatory lesion was not confined to any one part of the respiratory apparatus—it was not limited to the bronchial tubes as in simple bronchitis, nor to the pulmonary alveoli as in acute lobar pneumonia. The tubes, the peribronchial tissue, the alveoli, the interstitial tissue, and the pleural membrane, were all involved; thus the universal character of the inflammatory lesion, as well as the bacteriology of the disease, indicates that in acute infantile broncho-pneumonitis we are dealing with a simple rather than a specific inflammation of the lung. In all the cases differential counts of the blood corpuscles were made, in all a leucocytosis was demonstrated, and all the three cases terminated fatally, which is a matter to be noted, for it has been held by some that a leucocytosis, if at all marked, is of favourable import as regards prognosis.

Acute infantile pneumonitis is usually, he says, sudden in its onset. The infant may have been suffering before, but when the more serious disease begins the symptoms at once assume a more urgent and grave character. High fever sets in and the child soon becomes prostrate, the respirations become rapid and laboured, the alæ nasi dilating actively with every imspiration. Cough may not be very urgent, sometimes it is suppressed, and generally it is not very vigorous or paroxysmal. There may be diarrhœa or there may be constipation and tympanitic distension of the abdomen. Marked cyanosis or pale lividity soon sets in. The physical signs meanwhile are often rather indefinite. There may or may not be generalised bronchial wheezing, but what does catch the ear on auscultation is the sound of sticky medium or fine crackling, or subcrepitant râles on inspiration, which though fairly generally distributed tend to linger more persistently in particular areas of the lungs. Sometimes small patches of dulness may be detected, some of which may be large enough to suggest a lobar pneumonia or a pleural effusion; but on the other hand there may be no dulness at all. The occurrence of empyema should always be suspected if the febrile symptoms show no tendency to abate after a week or ten days, if the dulness persists over a single spot, and if with some alleviation of the more severe general symptoms an irregularly remitting type of fever sets in, accompanied by sweating. When pus has formed and been found the case should be dealt with surgically at once; such cases usually do well.

[1] Lancet, Sept. 20.

